# Annotations

**Published:** 1892-03-12

**Authors:** 


### Annotations.

**The Care of the Feeble-Minded.**

The Charity Organisation Society is becoming practical instead of merely inquisitorial. This is as it should be; and all kindly-disposed persons will be pleased to note an improvement in its morals. A deputation from the society, consisting of the Rev. Brooke Lambert, Mr. Timothy Holmes, F.R.C.S., Mrs. M'Callum, and Mr. C. S. Loch, waited upon Mr. Ritchie at the Local Government Board to urge the desirability of better provision for the care of feeble-minded women and girls. This is eminently a step in the direction of a higher humanity. In an ideal state, such as one would like England to be, all persons who could not take care of themselves would be taken care of, and all who could only help themselves a little would have their efforts at self-help supplemented by kindly aid from regulated benevolence. The feeble-minded, especially among women and girls, are in a very unhappy plight. At first sight it might seem that a cripple, or a deaf or blind person is much worse off than another who is in possession of all his bodily powers, and whose mind only is feeble. But this is by no means the case, as a little reflection will show. Let a man, or a woman, or even a child be but endowed with a high and resolute spirit and there are hardly any difficulties which cannot be overcome. Cripples of all kinds have become famous at many things; and it would not be difficult to mention the names of both deaf and blind persons who have accomplished far more than ordinary folks in the full possession of all their senses. But whoever heard of a feeble minded man or woman doing anything great, or indeed anything at all, except a little routine work of the most mechanical and least intellectual kind? As a matter of fact the feeble-minded always become either the care of those who are more intelligent than themselves, or the prey of those who are more vicious. The members of the deputation that waited upon Mr. Ritchie, and their supporters, are animated by a worthy anxiety to provide against the brutalisation and degradation of the feeble-minded by securing for them the help and protection of the public authorities. It is desired that Boards of Guardians, which are already endowed with permissive powers for placing blind, deaf, or dumb children in suitable institutions, shall have similar powers conferred upon them to deal with the feeble-minded, in concurrence with qualified medical practitioners. Mr. Ritchie put his finger upon one very obvious objection to the proposal of the deputation. One or two matters, he said, would have to be carefully weighed, and among other things they would have to consider how far it was desirable to keep feeble-minded children separate from their more fortunate fellows. That, of course, is a detail, though an important one. The answer to Mr. Ritchie's criticism is that feeble-minded children must be placed under the care of kind and intelligent instructors, and must be given such an education and such a training in arts and crafts as they are found to be most fit for. They will thus be rendered not only as happy as they can be made, but also far more useful, as well as much less costly to their natural guardians and the public exchequer, than if left to fight a losing battle against their more highly-endowed competitors.

**A Little Bacteriology.**

It is plain that we have by no means sounded the ultimate depths of bacterial knowledge and possibility. Whether or not we ever shall do so it would be rash even to speculate. Nobody ever thought that the science would be amusing until Dr. Hunter appeared upon the scene. Dr. Hunter reminds us of Lord Macaulay, of whom a famous statesman once said that, "If he could be as cock-sure of any one thing as Tom Macaulay was of everything, he could live and die content." Dr. Hunter, no doubt, owes his confidence to his northern blood, and to the splendid oxidising properties of the Edinburgh climate. As for poor Dr. Klein, the depressing fogs of London have made him sceptical, and he prefers to speak "with bated breath," if not "with whispering humbleness." Dr. Hunter has a splendid faith in phagocytes. According to him they are noble soldiers, who meet the enemy at the very gate of the city, and fight him inch by inch until they are driven to the citadel of the spleen. Not soldiers, says Dr. Klein, but scavengers are these same unheroic phagocytes. The real defenders of the body are dissolved in the liquid parts of the blood; and it is only when they have killed the invading microbe that the phagocyte comes forward and claims the dead body as his peculiar perquisite. The outsider can but look on with awe whilst all these wonders are being done, and with still greater awe whilst they are being learnedly talked about. In the meantime a word of comfort comes from the waning splendours of Berlin. It is not on this occasion a bespectacled German who proclaims to a listening world the mysteries of his laboratory. It is Dr. Kitasato, one of those wonderful Japanese, who having first imbibed the milk of science at the breasts of a Western mother, has suddenly shot up into a scientific giant, and essays to take the whole Western world by storm. Kitasato thinks he has discovered a fact which is of comfortable omen. According to him, the bacillus tuberculosis which is found in the expectoration of consumptive patients, and which for the last few years has been held to constitute so tremendous a danger to those who live near, or nurse consumptives, is not at all deadly to others, but is, on the contrary, itself actually dead. Not only are the bacilli that are found in the expectoration dead, but even those that are lying in the cavities of phthisical lungs are dead also. This discovery, if it should prove a discovery, upsets some theories of a very definite kind. But however hardly it may bear upon certain experimental physiologists, it is undoubtedly the most comfortable word which has issued from the physiological laboratory for some time.

**A Boy Murderer.**

The boy John Wise, aged seventeen years, who deliberately pushed a companion over a cliff at Portland without provocation, and with the object of killing him, in November last, has been declared, on medical evidence to be of weak and ill-balanced mind, and subject to mental aberration. This is right and proper so far as John Wise himself is concerned, but it will not bring back his murdered companion to life or allay the grief of surviving relatives. The case is one which compels attention to a much-neglected but very important subject. A congenital tendency to crime is the inheritance of a considerable proportion of our population. The class of persons who are afflicted with such a tendency should be recognised, if possible, before they have done any serious mischief, and discriminately dealt with. It would have been the easiest thing in the world for the most ordinary expert to have discovered that John Wise was not a sane boy long before he pushed his companion over the fatal cliff. Evidence was given which showed that he had declared some months before the sad crime that he had murdered a boy at Croydon. His statements, on investigation, were found to be false. But the very fact that a boy of that age, and of compulsorily sober habits, could make such a statement rendered it evident that his mind was running upon murder. Moreover, there were other facts disclosed which made it quite obvious that the boy was not like ordinary youths of his age, but had a mental organisation of a totally different character. What ought to have been done with John Wise when his peculiarities became known was that he should have been taken to an expert. Such an expert would have declared him to be a boy of homicidal tendency. He could then have been placed under careful control, and set to work whereby he could have earned his own living without risk to the life of a helpless companion, and without himself becoming irresistibly, a murderer in actual fact. The want of a little elementary scientific knowledge on the part of the public authorities responsible for these two boys has resulted in the tragic death of one of them, and in the worse than death of the other. Of the two, John Wise is the more to be pitied. He is clearly mad, and his madness takes the form of an uncontrolable impulse to kill. He, of course, will be placed in circumstances where his impulses will be watched and kept in check. But there are probably some scores of other boys, as well as men and women, at large, who, every now and again, find themselves the victims of an almost resistless desire to slay some of their fellow-creatures. It may be difficult in private life to put the finger of authority upon such dangerous persons, but in all schools and other institutions where human beings are under public supervision, it would be quite easy to separate the dangerous from the harmless of both sexes. What we wish to insist upon is that science has placed at our disposal the means of separating the safe and the unsafe among us into definite classes; and we ought, therefore, as rightminded custodians of knowledge and power, to hold it a duty to prevent murder and other dangerous crimes in all possible cases, as well as to control the insane murderer after he has done his dreadful deed.

